# Isolated Brainstem Involvement in a Patient with Hypertensive Encephalopathy

**DOI:** 10.1155/2013/540947

**Published:** 2013-02-28

**Authors:** Y. Osman, Y. Z. Imam, K. Salem, H. Al-Hail, B. Uthman, D. Deleu

**Affiliations:** ^1^Department of Neurology and Neurophysiology, Hamad Medical Corporation, Doha, Qatar; ^2^Department of Radiology, Hamad Medical Corporation, Doha, Qatar; ^3^Weill Cornell Medical College in Qatar, Doha, Qatar

## Abstract

Hypertensive encephalopathy typically presents with headache, confusion, and bilateral parietooccipital vasogenic edema. Brainstem edema in hypertensive encephalopathy usually occurs in association with typical supratentorial parieto-occipital changes and is usually asymptomatic. We report here a patient with hypertensive encephalopathy, with isolated brain stem involvement on magnetic resonance imaging (MRI). Rapid treatment of hypertension resulted in clinical and radiological improvement. Prompt recognition of the condition and aggressive treatment of hypertension in such patients is crucial to relieve edema and prevent life-threatening progression.

## 1. Introduction

 Hypertensive encephalopathy (HE) is a clinical-radiographic syndrome of heterogeneous etiologies occurring in about 16% of patients with hypertensive crisis [1].

Computed tomography (CT), or magnetic resonance imaging (MRI) in hypertensive encephalopathy patients often shows bilateral white matter edema of the parieto-occipital lobes. The term posterior reversible encephalopathy syndrome (PRES), also known as reversible posterior leukoencephalopathy syndrome (RPLS) [2], was first described by Hinchey et al. in 1996. They described a clinical syndrome of insidious onset of headache, confusion or decreased level of consciousness, visual changes, and seizures. Brainstem lesions are associated with cerebral posterior white matter changes in 58% of patients [3]; however, it is rare for hypertensive encephalopathy to have isolated involvement of the brainstem without concomitant parieto-occipital lesions [3, 4].

 Magnetic resonance imaging (MRI) usually reveals symmetric, often reversible, hyperintense lesions on T2-weighted images suggestive of vasogenic edema. We report here a patient with hypertensive encephalopathy presenting with isolated brainstem edema without concomitant parieto-occipital edema.

## 2. Case Report

 A 32-year-old Qatari male, with a history of hypertension for over 2 years, presented to the emergency room with a witnessed generalized tonic-colonic seizures for 2-3 minutes. It was associated with loss of consciousness and tongue biting.

His past medical history includes Diabetes Mellitus (type 1) diagnosed at 9 years of age and end-stage renal failure secondary to advanced diabetic nephropathy which was treated with continuous ambulatory peritoneal dialysis for the last 2 years. He was maintained on insulin and anti-hypertensive medications, notably fosinopril 20 mg once daily and nifedipine retard 40 mg once daily.

One day before developing the seizures, the patient experienced headache and dizziness, but no fever.

On arrival to the emergency room he was confused and irritable with a blood pressure of 220/140 mm hg, a pulse rate of a 100 beats/min, and a body temperature of 37.1°C. His blood chemistry is shown in Table 1.

The complete blood count (CBC), the liver function tests, and the serologic screening for vasculitis were normal. The serum thiamine level was within normal limits. Because the patient was agitated, a detailed neurological examination was not performed initially.

Later on the patient became unable to maintain his airways and was intubated. Immediately an intravenous infusion of labetalol was started.

**Figure 1:**
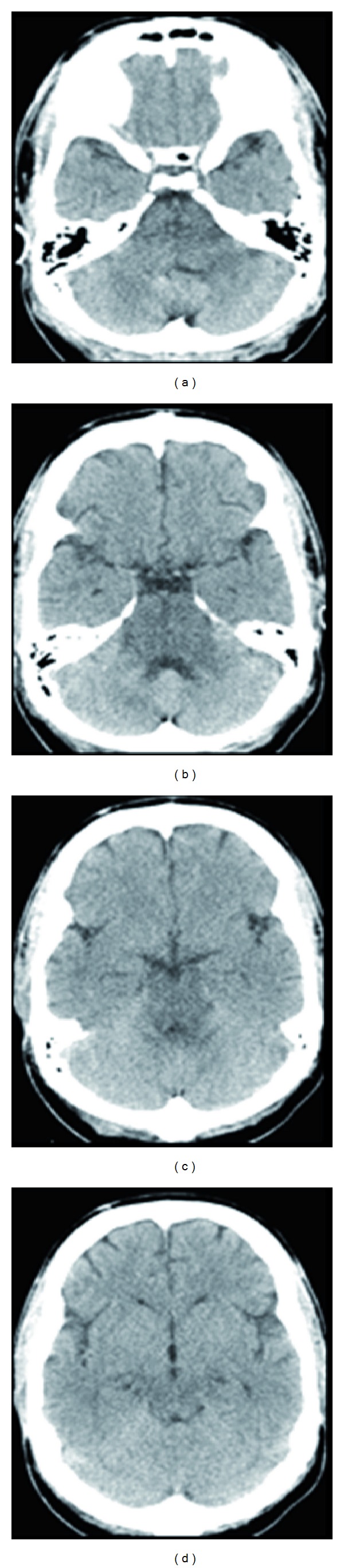
Noncontrast brain CT scan showing isolated hypodensity of the whole Pons ((a) and (b)) and midbrain ((c) and (d)) with narrowed prepontine, cerebellopontine, and ambient cisterns.

An urgent non-contrast CT scan of the brain was done 4 hours after the admission. It showed diffuse brain stem hypodensity (Figure 1). Phenytoin 300 mg per nasogastric tube (NGT) was added.

An EEG was performed, which showed diffuse, bilateral slowing in the theta, and delta frequency range, without any evidence of active discharges.

**Figure 2:**
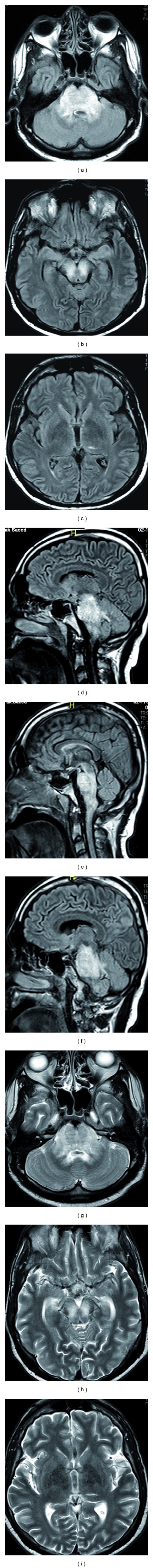
Noncontrast MRI showing swollen Pons with isolated high signal intensity of the whole Pons and midbrain in fluid attenuated inversion recovery (FLAIR) axial ((a)–(c)), sagittal ((d)–(f)), and T2-weighted (T2WI) ((g)–(i)) images.

**Figure 3:**
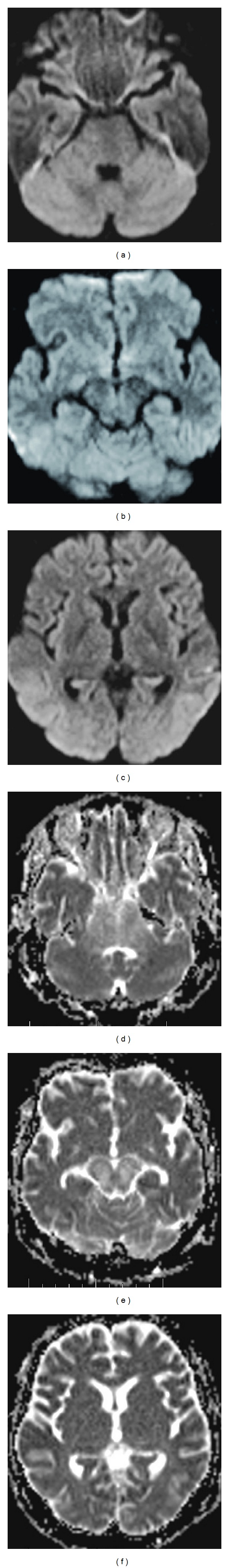
Magnetic resonance imaging (MRI) diffusion-weighted images (DWI): axial images ((a)–(c)) showing no restricted diffusion, and ADC map images ((d)–(f)) with increased apparent diffusion coefficient in the brain stem.

Magnetic resonance imaging was conducted 48 hours after the admission, which revealed swollen brainstem with isolated central and peripheral high signal in the Pons, midbrain, and middle cerebellar peduncles on the fluid attenuated inversion recovery (FLAIR) and T2-weighted images (T2WI) (Figure 2); normal intensity on diffusion-weighted images (DWI); and increased values of apparent diffusion coefficient (ADC map) (Figure 3). The same area showed T1WI isointense signal but no contrast enhancement. The intracranial magnetic resonance angiography (MRA) was normal. Tightness of the basal cisterns and posterior fosse CSF spaces were noted; however no tonsillar herniation was seen.

The MRI features were consistent with vasogenic edema, involving the whole brainstem likely indicating hypertensive encephalopathy.

The blood pressure was aggressively and successfully treated with intravenous infusion of labetalol. The systolic blood pressure was lowered to 180–150 mm Hg and the diastolic blood pressure to 110–100 mm Hg. Later on, long acting nifedipine 40 mg once daily per NG tube was added. His blood pressure continued to fluctuate during the first 4 days of admission and then fosinopril 20 mg once daily was restarted.

Cerebrospinal fluid study showed normal opening pressure of 130 mm H_2_O, a white blood cells count 2 cells/mm^3^, a normal CSF-glucose level of 9.50 mmol/L (normal 60–80% of blood glucose), and a normal cytological exam. However the CSF protein was elevated at 0.97 g/L (normal 0.15–0.45 g/L).

 Bacterial, fungal, and mycobacterial stains and cultures were negative. An electrocardiograph (ECG) showed left ventricular hypertrophy, and a transthoracic echocardiogram showed concentric left ventricular hypertrophy with no evidence of intracardiac masses or thrombi. 

The sedation was tapered off gradually and was turned off by the third day of admission. Neurological examination at that time showed that he was drowsy, but opened his eyes spontaneously and responded to simple verbal tasks. The pupils were bilaterally equal-sized and reactive to light. There was normal range of ocular movement. He could move all 4 limbs equally. There were exaggerated deep tendon reflexes over both legs and extensor plantar responses bilaterally. 

During the second week of admission his blood pressure started to stabilize and there was a significant improvement in his mental status.

**Figure 4:**
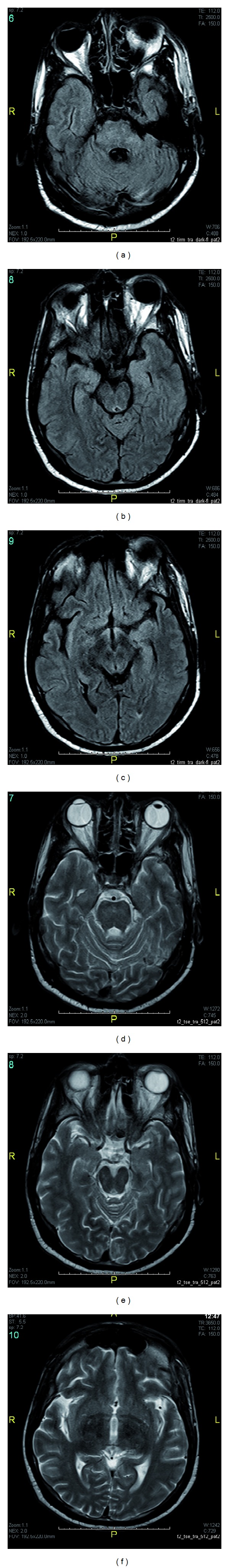
Follow-up non-contrast MRI after 12 days showing significant regression of the brain stem edema bright signal in FLAIR axial ((a)–(c)) and T2WI ((d)–(f)) images.

A repeated MRI-brain was done 12 days after admission, which revealed marked improvement with a significant reduction in the brainstem edema (Figure 4).

The patient was gradually weaned off the ventilator support and was extubated after 17 days of admission. 2 days later he was transferred to the general medical ward, where mobilization with support was initiated. He remained conscious and fully orientated. Phenytoin was stopped on day 25. The patient was discharged on day 39, after establishing mobilization without support.

**Figure 5:**
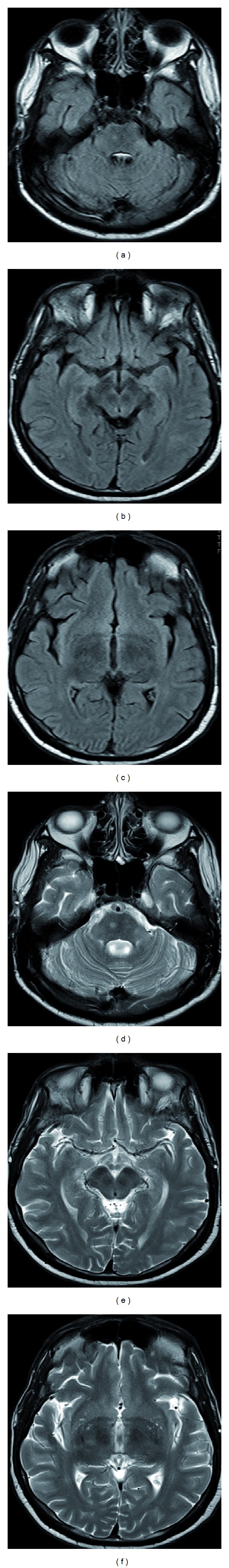
Follow-up non-contrast MRI after 2 months showing almost total resolution of the brain stem previous bright signal in FLAIR axial ((a)–(c)) and T2WI ((d)–(f)) images apart from tiny residual bright spot on right side of the Pons.

A follow-up brain MRI was done after 2 months, which showed almost total resolution of the brainstem bright signals and swelling in FLAIR, and T2WI, apart from tiny residual spot on the right side of the Pons (Figure 5).

## 3. Discussion

 Hypertensive encephalopathy results from acute elevation of blood pressure beyond the upper limits of cerebral autoregulation [2]. The neurologic syndrome in hypertensive encephalopathy is now believed to be caused by vasogenic edema from the breakthrough of the autoregulation [5].

Although hypertensive encephalopathy is described in malignant hypertension alone, the similar clinical and radiological manifestations can be seen more common in patients with comorbid conditions, such as systemic lupus [6], cryoglobulinemia [7], or hemolytic-uremic syndrome [8], and in patients treated with cyclosporine [9] or cisplatin [10].

In healthy subjects, cerebral autoregulation is maintained by both myogenic and neurogenic components, with the latter being dependent on the degree of sympathetic innervations. In patients with hypertensive encephalopathy, passive overdistension of the blood vessels due to rise in blood pressure blunts the myogenic response. The neurologic component thus predominates and there is breakdown of the blood-brain barrier with focal transudation of fluid and proteins into the brain interstitium.

The predilection for involvement of posterior circulation territories is generally accepted to result from the relatively sparse sympathetic innervations of the vertebrobasilar circulation [11].

Neuroimaging is essential for the diagnosis of hypertensive encephalopathy. Typical findings are symmetrical white matter edema in the posterior cerebral hemispheres, particularly the parieto-occipital regions, but variations do occur [12].

Neuroradiographic abnormalities of hypertensive encephalopathy are often apparent on CT scans, but best demonstrated on MRI. The most commonly observed abnormalities on MRI are punctuate or confluent areas of increased signals on FLAIR and T2-weighted images [13]. Diffusion-weighted images (DWI) aids in the distinction of hypertensive encephalopathy from top of the basilar stroke. Vasogenic edema is usually visualized as a hypo- or isointense signal on DWI and increased signal on apparent diffusion coefficient (ADC) maps [14]. This is in contrast to acute cerebral infarction which produces marked hyperintensity on DWI and hypointensity on ADC maps. However, atypical MRI findings such as infarction with permanent residual lesions and hemorrhage are also reported [15].

The clinical manifestation of hypertensive encephalopathy is characterized by headache, altered consciousness, visual disturbance, and seizures [12].

We describe a case with clinical and imaging findings, which occurred with elevation of blood pressure and resolved after several days following the normalization of the blood pressure. 

Seizures disorders are the presenting manifestation as in ourpatients [16]. Seizures are usually generalized tonic clonic. Most patients with hypertensive encephalopathy and seizures are treated with phenytoin [17]. Phenytoin then can be safely tapered off as symptoms and neuroimaging findings resolve in two weeks upon average [18].

Hypertension is a feature in the majority of patients with hypertensive encephalopathy, regardless of the etiology [2]. In our patient the high blood pressure was successfully treated with intravenous labetalol, with lowering of the diastolic blood pressure to about 100–105 mmHg, within the first six hours. After stabilization of the blood pressure, a few days later, the patient had improved dramatically.

Brainstem involvement is not infrequent, but is commonly associated with the more typical parieto-occipital changes [4], which were absent in our patient. Isolated involvement of the brainstem is rare, with a few cases in the literature [19].

End-stage renal disease is the risk factor in this patient. In one review by Cruz-Flores, et al. [4], two-thirds of the hypertensive encephalopathy patients with brainstem involvement, in addition to elevated blood pressure, had comorbidities such as renal failure. An explanation would be that the vasoconstriction response fails to maintain constant blood perfusion during hypertension in the uremic state, as demonstrated in the animal model [20].

The most important differential diagnosis in this patient is massive brainstem infarction, because the goals of blood pressure treatment are different. The MRI evidence of vasogenic edema, that is, absence of high signal lesions on diffusion weighted images and increased value of apparent diffusion coefficient, is strongly diagnostic of hypertensive brainstem encephalopathy [2], but not infarction. Additionally the MR angiography revealed no stenosis of the vertebrobasilar arteries. Furthermore, our patient had only seizures and drowsiness, but had no ophthalmoplegia or quadriparesis, as might be expected from the extensive brainstem abnormalities seen on the CT and MRI. 

Morello et al. had reported a patient with hypertensive brainstem encephalopathy, who had clinically silent massive edema of the Pons [21]. This clinicoradiographic dissociation is characteristic of hypertensive brainstem involvement [4] and could be explained by reversible vasogenic edema in hypertensive brainstem encephalopathy, but not irreversible cytotoxic edema and neuronal death as in brainstem infarction. The drowsiness could be explained by involvement of the brainstem reticular activating system.

The brainstem CT and MRI findings in our patient should also be differentiated from diffuse glioma, infectious brainstem encephalitis, Wernicke's encephalopathy, and central pontine myelinolysis. The marked and rapid clinical and radiological improvement, after only normalization of the blood pressure, excludes glioma. The lack of fever and other signs of infection and the lack of CSF pleocytosis make infectious encephalitis unlikely. Wernicke's encephalopathy was ruled out, because there were no opthalmoplegia or amnesia, and no increased signal in periaqueductal regions and medial thalami. Moreover, the blood thiamine level was normal. 

The reversibility of symptoms and imaging findings coupled with a negative history of rapid correction of hyponatremia makes central pontine myelinolysis less likely. The absence of prior infection, vaccination, external ophthalmoplegia, and subcortical white matter changes makes the possibility of acute disseminated encephalomyelitis (ADEM) less likely.

Prominent CSF protein elevation is uncommon in hypertensive brainstem encephalopathy, but uremia often increases the CSF protein. CSF protein elevation >60 mg/dL occurs in 60% of uremic patients. Moreover CSF protein exceeds 100 mg/dL in 20% of uremic patients [22]. 

## 4. Conclusion

Isolated brainstem hypertensive encephalopathy is rare. It is characterized by clinicoradiographic dissociation. Prompt and rapid treatment of hypertension is important to reverse posterior fossa edema and potentially life-threatening complications.

## Figures and Tables

**Table 1:** 

	Patient's value	Normal range
Blood sugar	27 mmol/L	3.3–6 mmol/L
Chloride	92 mmol/L	96–110 mmol/L
Potassium	5 mmol/L	3.6–5.1 mmol/L
Sodium	129 mmol/L	135–145 mmol/L
Urea nitrogen	29.1 mmol/L	1.7–8.3 mmol/L
Creatinine	967 mmol/L	62–124 mmol/L
Bicarbonate	19 mmol/L	22–29 mmol/L
Osmolarity	317 os/kg	275–295 mmol/L
Corrected	Calcium 1.9 mmol/L	2.1–2.6 mmol/L

Ketone bodies were not detected.
